# Serum Biomarkers in Atlantic Salmon for Differential Diagnosis of Cardiomyopathy Syndrome and Pancreas Disease: Proteomic Identification of Serum Fibrinogen to Enhance Troponin Immunoassay as Optimal Diagnostic Approach

**DOI:** 10.1111/jfd.14151

**Published:** 2025-05-29

**Authors:** Francesca Riva, Lauren Elaine Black, Kim D. Thompson, Philippe Sourd, Andrei Bordeianu, Christopher C. Chadwick, Hooman Moghadam, Jorge Del‐Pozo, Janina Costa, Richard Burchmore, Nicola Brady, Lewis Moore, Suzanne McGill, Mark McLaughlin, Peter David Eckersall

**Affiliations:** ^1^ School of Biodiversity, One Health and Veterinary Medicine University of Glasgow Glasgow UK; ^2^ Moredun Research Institute Penicuik UK; ^3^ Cooke Aquaculture Scotland Ltd. Bellshill UK; ^4^ Life Diagnostics Inc. West Chester Pennsylvania USA; ^5^ Benchmark Genetics Norway AS Bergen Norway; ^6^ Royal (Dick) School of Veterinary Sciences University of Edinburgh Roslin UK

**Keywords:** Atlantic salmon, biomarkers, cardiomyopathy syndrome, fibrinogen, pancreas disease

## Abstract

Cardiac viral diseases are among the major causes of economic losses in Atlantic salmon ( *Salmo salar* L.) aquaculture. These include cardiomyopathy syndrome (CMS) caused by piscine myocarditis virus (PMCV) and pancreas disease (PD) caused by Atlantic salmonid alphavirus (SAV). The resulting cardiomyopathies impact fish stock in terms of mortality, quality, growth performance and economic loss. Diagnosis of these diseases is currently based on clinical signs, histopathology and RT‐qPCR. To identify putative biomarkers for use in the health assessment of Atlantic salmon, a quantitative proteomics investigation was undertaken with the aim of differentiating fish with CMS from healthy fish and fish with PD. Serum samples (*n* = 9/group) were collected during health assessment from pens where clinical CMS or PD were present and compared to serum from healthy Atlantic salmon. There were 34 differentially abundant proteins (DAPs) in CMS compared to healthy, 66 comparing CMS to PD, and 81 comparing PD to healthy. In relation to healthy samples, most DAPs were shared between CMS and PD, with higher relative abundances observed in PD. An exception to this was serum fibrinogen, which was identified as a putative biomarker for CMS, whereas differentiation of Atlantic salmon with CMS from those with PD was enhanced by the calculation of the ratio of fibrinogen to skeletal troponin C.

## 1 Introduction

Among health challenges in aquaculture, viral diseases of Atlantic salmon include infections with Piscine myocarditis virus (PMCV) causing cardiomyopathy syndrome (CMS) (Garseth et al. 2018), Atlantic salmonid alphavirus (SAV) causing pancreas disease (PD) and Piscine orthoreovirus (PRV) causing heart and skeletal muscle inflammation (HSMI) (Costa et al. 2022). Developing and improving methods to monitor and respond with appropriate health management to these clinical or subclinical diseases in farmed Atlantic salmon populations is highly desirable and especially in their differential diagnosis, which is currently based on histopathology and other laboratory methods at a distance from farm sites that are not conducive to providing rapid diagnosis. The pathophysiology associated with PD, particularly its effects on skeletal and cardiac muscles, leads to significant alterations in serum biochemistry with notable elevations in the activity of muscle enzymes such as creatine kinase and enolase (Braceland et al. 2015). It has recently been shown that the biomarkers cardiac troponin (cTnC) and skeletal muscle troponin (skTnC) are elevated during *Flavobacterium psychrophilum* infection in trout determined by immunoassay using antibody raised to the salmon troponins (Wiens et al. 2023). Identifying additional biomarkers for PD and CMS in Atlantic salmon would enhance diagnostic and monitoring systems during production. Biomarkers indicative of muscle damage could provide valuable insight into disease progression, particularly if they are elevated during CMS and PD. Moreover, distinguishing between the two conditions is crucial, and it would enhance the development of rapid immunoassay‐based diagnostics that could be deployed on farm. Novel biomarkers can be identified by proteomics, a technology that enables the comprehensive study of all proteins in a sample, which in turn enables the search of putative serum biomarkers for early detection of major diseases affecting Atlantic salmon aquaculture. The serum proteome of Atlantic salmon during PD and CMS has been previously studied to assess changes following infection (Costa et al. 2021). The study of Costa et al. (2021) identified proteins associated with cardiac disease in humans, categorised as leakage enzymes, host reaction proteins and regeneration/remodelling proteins. While these findings were promising, Costa et al. (2021) did not establish these proteins as definitive biomarkers for CMS. In contrast, serum enolase activity has been successfully identified as a potential biomarker for PD using a proteomic approach (Braceland et al. 2013, 2015). Similar approaches have also been employed to identify candidate biomarkers in gill disease (Marcos‐Lopez et al. 2017). Overall, the use of proteomics for biomarker discovery has seen previous application in aquaculture research (Jaiswal et al. 2023), supporting its value in the development of novel diagnostic tools. An unexpected finding in the previous proteomics study (Costa et al. 2021) was that fibrinogen in serum was identified as a potential biomarker for CMS, despite the blood samples collected having been allowed to clot and the resulting coagulated fibrin removed during the preparation of serum. Conventionally, fibrinogen is assayed in plasma (Brooks 2022) to detect and monitor changes in coagulation, while serum is not considered to be a suitable sample type for fibrinogen analysis for the above reason. There have been few investigations of fibrinogen in salmon plasma, and indeed, this biomarker was not included in a recent study of 33 analytes of biochemistry and haematology in plasma from Chinook salmon (Owen et al. 2025). In one of the few investigations, there have been on fish fibrinogen, a study of tilapia following experimental infection with *Streptococcus iniae* showed that plasma fibrinogen assessed by a coagulation method decreased, whereas in terrestrial animals, fibrinogen would be considered an acute phase protein that increases following infection (Ceron et al. 2005). Among methods used for quantitative proteomics, tandem mass tags (TMT) have been used to identify potential biomarkers in mammalian species (Horvatic et al. 2019; O'Reilly et al. 2022; Turk et al. 2021). In this study, TMT‐proteomics has now been applied to identify biomarkers for CMS and for differentiation from PD by validation of proteomic results in combination with troponin immunoassays for the benefit of Atlantic salmon health, using serum samples collected during routine health monitoring across three farms over a 6‐month period.

## 2 Material and Methods

### 2.1 Sample Collection and Selection

Serum samples were collected from Atlantic salmon between 3 and 8 months post seawater transfer from three Cooke Aquaculture Scotland fish farms (A, B and C) during routine health monitoring carried out by aquaculture veterinary experts. Farms A and B historically had previous outbreaks of PD and CMS, while farm C experienced lower incidences of viral disease in comparison. Outbreaks of CMS and PD in Farms A and B were observed and noted by the aquaculture veterinary specialist performing the health assessment, which served as the basis for categorising disease presence in this study. Clinically, healthy fish samples were confirmed in Farm C where no disease was observed. During each farm visit, eight serum samples were collected from three study pens per farm. Following coagulation and centrifugation, sera samples were frozen at −20°C after collection, transferred on dry ice to laboratories for analysis and stored at −20°C until processed.

The CMS group samples in this study were taken from pens in a farm where a clinical CMS outbreak was confirmed in the population at that point in time. This was diagnosed by qPCR positivity for PCMV in samples of moribund fish taken from the same population (*n* = 4). These moribund fish presented with CMS signs such as ascites, acute mortality onset and cardiac lesions, including atrial rupture. Histological analysis of samples from these fish revealed chronic myocarditis in the spongy layer of the cardiac ventricle, with or without atrial thrombosis.

The PD samples were taken from pens in a farm that had clinical PD at that point in time. This was defined by qPCR positivity for SAV in cardiac tissue samples of clinically ill fish (*n* = 4) taken from the same population. These had clinical PD signs including sustained inappetence, weight loss and abnormal swimming behaviour. Measurements of conventional muscle damage biomarkers, such as creatine kinase (CK) and aspartate transaminase (AST), as well as concentrations of cTnC and skTnC, were also measured.

### 2.2 Serum Biochemistry Analysis

The enzymatic activity of the established biomarkers of muscle damage CK and AST in the Atlantic salmon serum samples was determined on a Dimension Xpand Plus biochemical analyser (Siemens Healthcare Ltd., Camberley, Surrey, UK) using reagents DF38 and DF41A, respectively, at the Veterinary Diagnostic Service, School of Biodiversity, One Health and Veterinary Medicine, University of Glasgow.

The concentrations of cTnC and skTnC in each sample were determined using spatial proximity analyte reagent capture luminescence (SPARCL) assays (Veterinary Biomarkers Inc., West Chester, USA), following the procedure and validation described previously (Wiens et al. 2023) and further confirmed at Life Diagnostics (West Chester, USA). For assessment of precision of the skTnC assay, four serum samples from PD‐diagnosed salmon were tested in triplicate across three independent assays. Each run employed fresh sample dilutions (1:500), conjugates and standard curves. The average intra‐assay coefficient of variation (CV) was 1.4%, and the inter‐assay CV was 3.5%. Dilutional linearity was demonstrated using three samples tested at dilutions ranging from 1:250 to 1:4000, confirming consistent performance of the assay. For assessment of the precision of the cTnC assay, serum from four CMS‐diagnosed fish was similarly analysed in triplicate across three separate assays using fivefold serial dilutions. Each experiment used freshly diluted serum, new conjugates and new standards. The intra‐assay CV averaged 2.3%, and the inter‐assay CV was 4.3%. Dilutional linearity was observed at dilutions of fivefold or greater.

Fibrinogen was determined using an ELISA (FIB‐23, Life Diagnostics Inc., West Chester, USA) according to the manufacturer's instructions. The ELISA uses affinity‐purified rabbit polyclonal antibodies generated against purified Rainbow trout fibrinogen; it recognises Rainbow trout and Atlantic salmon fibrinogen indistinguishably. Samples were initially pre‐diluted in assay buffer at 1:20,000, 1:40,000 and 1:80,000, with the 1:40,000 dilution being optimal. The mean CV of serum samples with concentrations of fibrinogen > 100 mg/mL (*n* = 21) analysed in triplicate was 11.9%.

### 2.3 Quantitative TMT Proteomics Investigation of Atlantic Salmon Serum

For the quantitative TMT proteomic analysis, nine serum samples per group from the CMS, PD outbreaks, and healthy controls were selected to assess differences in protein abundances. Proteomic analysis was performed by applying an 11plex TMT‐based quantitative gel‐free approach to evaluate the effects of CMS and PD samples on the Atlantic salmon serum proteome in comparison to healthy samples (*n* = 9 per group) following a method previously described (Nguyen et al. 2022). Briefly, 150 μg of total serum proteins from samples and internal standard (pool of samples) was diluted to a volume of 40 μL and subjected to filter‐assisted sample preparation, which included alkylation, trypsin digestion and labelling with specific TMT reagents. The TMT‐modified samples were analysed by high‐resolution LC–MS/MS using an Ultimate 3000 RSLCnano system (Dionex, Germering, Germany) coupled to an Orbitrap Fusion Tribrid mass spectrometer (MS, Thermo Fisher Scientific, Bremen, Germany). Acquired MS/MS spectra were analysed for protein identification and quantification using the SEQUEST algorithm implemented into Proteome Discoverer (version 2.3., ThermoFisher Scientific). A database search against *Salmo salar* FASTA files (downloaded from Uniprot database on 19 December 2022, 48,387 sequences) was performed, requiring at least two unique peptides and 5% false discovery rate (FDR) for the confident reporting of identified proteins. Protein quantification was accomplished by correlating the relative intensities of reporter ions extracted from tandem mass spectra with those of the peptides selected for MS/MS fragmentation.

### 2.4 Analysis of Proteomic Data

Principal component analysis (PCA) was conducted using the factoextra package (Kassambara and Mundt 2020) within the R Studio environment using all proteins from healthy, CMS and PD groups. The individuals from principal components that accounted for most of the variation in the data were plotted in a graph, which also included the mean and 95% confidence interval.

For assessment of all proteomics data, the fold change (FC) and log_2_FC between two groups were calculated from protein abundance values, alongside the *p*‐value using a two‐sample Student's *t*‐test in R. Log_2_FC and *p*‐values were used to identify differentially abundant proteins (DAP) and to create volcano plots in R. DAPs were further analysed by creating heatmaps in R to investigate protein differences between groups. Additionally, GraphPad Prism 10 was used to graph specific proteins of interest from both proteomics and biochemistry data.

For functional analysis, the protein GenInfo Identifier (GI) accession numbers of DAPs from all three comparisons were inputted into The Database for Annotation, Visualisation and Integrated Discovery (DAVID) (Sherman et al. 2022). The direct Gene Ontology (GO) terms were assessed to understand gene enrichment with respect to biological process (BP). In addition, functional annotation clustering was performed with medium classification stringency, and KEGG pathway assessment was also carried out.

### 2.5 Validation of Proteomic Results

The proteomic results were validated by use of alternative assay procedures to determine whether similar results would be obtained. For validation through comparison with serum biochemistry analytes, the activity or concentration of CK, AST and fibrinogen in the Atlantic salmon serum samples was determined by assays described in Section 2.2 above. The ratio of the total fibrinogen concentration to the concentration of skTnC was also determined to augment the differential between CMS and PD or healthy concentrations, and for ease of interpretation, the concentration of fibrinogen was converted to mg/mL to compare to the skTnC concentration measured as ng/mL.

Western blot analysis was performed for fibrinogen as previously described (Braceland et al. 2015), with sample loading at either 2 or 0.2 mg of total protein per electrophoresis well and using antigen‐affinity‐purified rabbit polyclonal antibody to trout fibrinogen (Life Diagnostics, West Chester, USA) as the first antibody at a dilution of 1:40,000 and goat anti‐rabbit IgG conjugated to HRP (AbCam, Cambridge, UK, #ab6721) at a dilution of 1:5000 as the secondary antibody.

The effects of the viral infections on lipoprotein metabolism were determined as described previously (Nguyen et al. 2023) by investigating the cholesterol content of high‐density lipoprotein (HDL‐chol) and low‐density lipoprotein (LDL‐chol). This was performed using a method based on precipitation of the LDL fraction and measuring the cholesterol concentration in the supernatant as the HDL‐chol fraction using Cholesterol Assay Kit—HDL and LDL (Abcam Ltd. Cambridge UK, ab65390) with a clinical biochemical analyser (Dimension Xpand Plus, Siemens, Camberley, Surrey, UK). The LDL‐chol was determined by the Friedewald equation, which required the measurement of total cholesterol and triglyceride in the serum samples.

### 2.6 Statistics

Descriptive statistics and significance of differences between groups were determined by ANOVA, using the Kruskal–Wallis test and Dunn's multiple comparisons test for post hoc analysis in GraphPad Prism, v9.3.1.

## 3 Results

### 3.1 Serum Biochemistry Analysis

**FIGURE 1:**
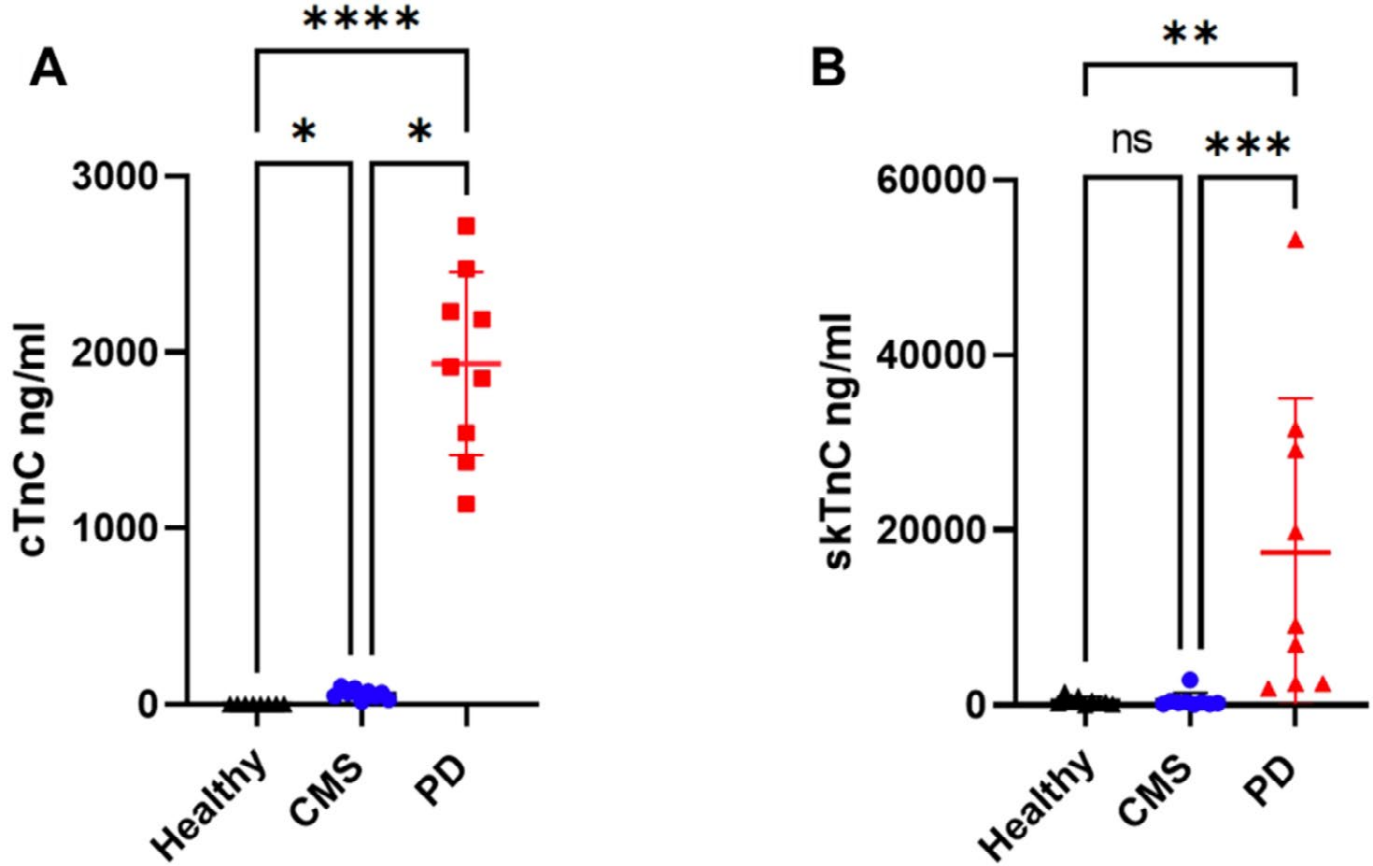
The concentrations of cTnC (A) and skTnC (B) in serum from healthy Atlantic salmon (*n* = 9) and those with CMS (*n* = 9) or PD (*n* = 9) as determined by SPARCL immunoassay. Differences between groups were determined by Kruskal–Wallis and Dunn's multiple comparison test, using Graphpad Prism version v9.3.1 *p < 0.05, **p < 0.01, ***p < 0.005, ****p < 0.0001.

Biochemical analysis of serum samples (Table 1, Table S1) revealed significantly elevated concentrations of both cTnC and skTnC along with increased enzymic activities of CK and AST in the PD group compared to both the CMS and healthy samples (Table 1, Figure 1A,B). When comparing CMS to healthy fish, cTnC was the only biomarker that showed a significantly higher concentration in the CMS group, with 58 ± 28 ng/mL (mean ± SD) compared to the healthy samples of 0.41 ± 0.29 ng/mL. However, this concentration was markedly lower than those observed in the PD group, which averaged 1937 ± 520 ng/mL. No significant differences in skTnC, CK and AST levels were observed between the CMS and healthy groups. In contrast, when CMS was compared to PD samples, concentrations of cTnC, skTnC and AST were significantly lower in the CMS group.

**TABLE 1:** Serum biochemistry of serum samples from Atlantic salmon in the healthy, CMS and PD groups as determined by use of SPARCL immunoassay for cTnC, and skTnC and by enzymatic analysis for determination of the activities of CK and AST. Statistical significance was determined using the Kruskal–Wallis test and Dunn's multiple comparison test, with *p*‐values as indicated in the table.

Group	cTnC ng/mL (*n* = 9)	skTnC ng/mL (*n* = 9)	CK IU/L (*n* = 8)	AST IU/L (*n* = 8)
Healthy Mean ± SD	0.41 ± 0.29^a^	491 ± 474^a^	219 ± 382^a^	481 ± 214^a^
CMS Mean + SD	58 ± 28^b^	539 ± 884^a^	125 ± 108^a,b^	418 ± 214^a^
PD Mean + SD	1937 ± 520^c^,*	17,406 ± 17,642^b^,**	8928 ± 19,111^b^	4676 ± 4244^b^,**

*Note:* a,b,c Significant differences between groups at *p* < 0.05.

* Significant difference between groups at *p* < 0.01.

** Significant difference at *p* < 0.005 determined by ANOVA by the Kruskal–Wallis test and Dunn's multiple comparison test, using GraphPad Prism version v9.3.1.

### 3.2 Proteomics

**FIGURE 2:**
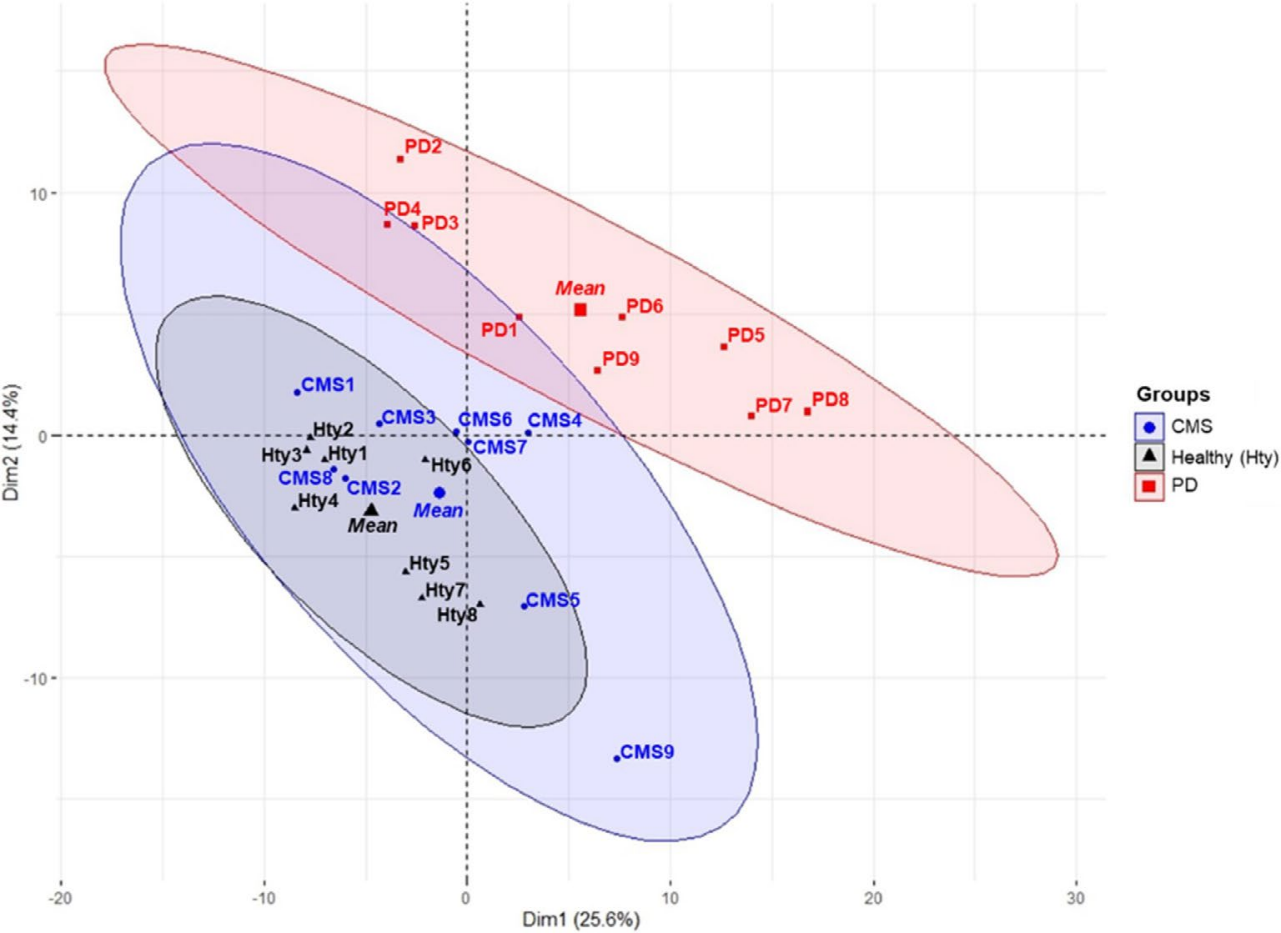
PCA plot with the two principal components that explained most of the variation in the data; the graph was produced to show the individuals in all groups. The elliptical clouds depict the 95% confidence interval for the means of each group. Group means are depicted by the larger symbols towards the centre of each ellipsis.

**FIGURE 3:**
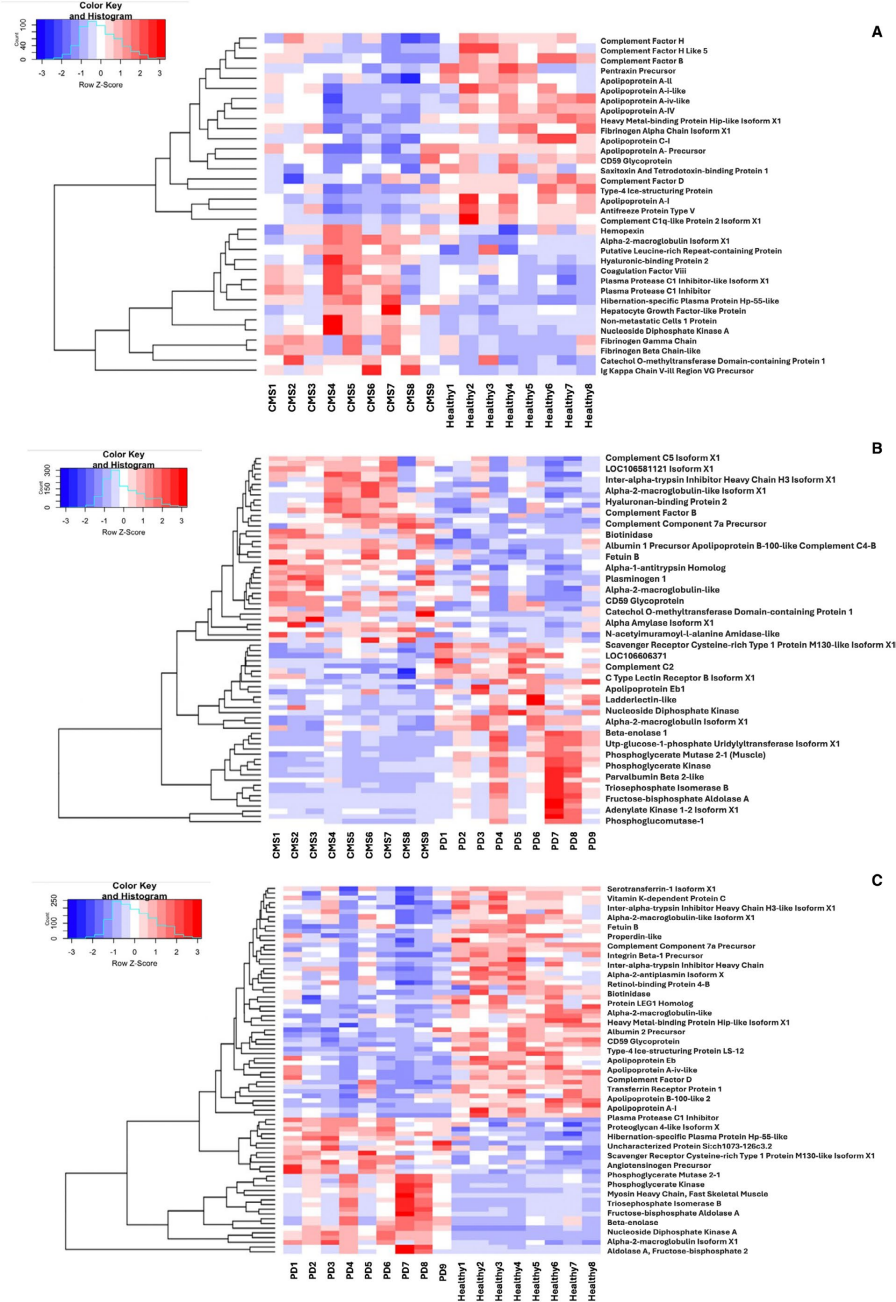
Heat maps of protein abundance in serum from healthy (*N* = 8), CMS (*N* = 9), and PD (*N* = 9) Atlantic salmon, generated in RStudio. (A) highlights proteins with significant differences (*p* < 0.05) between healthy and CMS Atlantic salmon, associated with unique genes. (B) illustrates the significant proteins between CMS and PD samples, while (C) shows the significant proteins between healthy and PD. In all figures, red intensity indicates a relative increase in protein abundance (as listed on the right), and blue intensity represents a relative decrease in protein abundance.

In the TMT proteomic analysis of the Atlantic salmon serum samples, one healthy serum sample failed to give adequate results in the mass spectrometer, leaving a total of eight samples for analysis. Overall, 674 proteins were identified, of which 255 had more than 1 unique peptide. When all protein abundance values were analysed by PCA (Figure 2), PD samples formed a separate cluster that did not overlap with the healthy or CMS groups, whereas the CMS cluster showed considerable overlap with the healthy cluster. Assessment of individual points revealed four CMS samples that were outside the healthy cluster and separate from the PD cluster (CMS4, CMS6, CMS7 and CMS9). Several DAPs were found in comparisons of samples from the CMS, PD and healthy groups, with a full list of proteomic results provided in Table S2 and uploaded to ProteomeXchange (submission reference: 1‐20241030‐131842‐123970160). In analysing the DAPs for potential biomarkers of CMS and PD, group comparisons were made by comparing heat maps of CMS and PD to healthy (Figure 3). The comparison of PD versus healthy samples revealed the highest number of DAPs (*n* = 81) the CMS versus healthy had the lowest DAPs (*n* = 34) and CMS versus PD was in between (*n* = 66).

In the comparison of CMS versus healthy samples, the volcano plot (Figure S1) displays all identified proteins, while the heatmap (Figure 3A) highlights the significant DAPs per each comparison group. Table 2 lists the five proteins with the greatest increase and decrease in FC in abundance, along with further proteins relevant to the search for disease biomarkers. Notable DAPs that were significantly higher in CMS compared to healthy samples included Ig kappa regions, b and g fibrinogen, nucleoside diphosphate kinase, plasma protease inhibitor C1 and hemopexin, while apo A‐IV, apoA1A, apo C1, a fibrinogen, complement factors H and B, and ice‐structuring protein type 4 showed decreased levels in CMS.

**TABLE 2:**
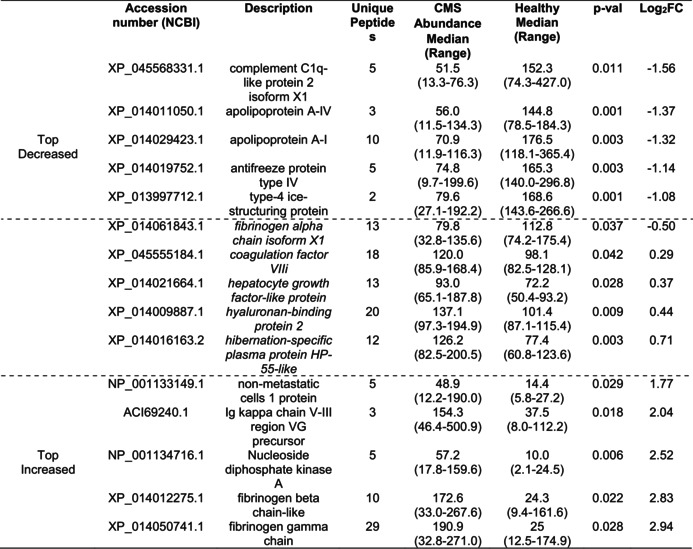
The five most decreased and five most increased differentially abundant proteins in comparison of samples from CMS to healthy Atlantic salmon, including in italics and between the dotted lines further proteins of potential biomarker applications.

For the CMS versus PD serum proteome comparison, the volcano plot (Figure S2) similarly shows all identified proteins, and the heatmap (Figure 3B) depicts significant DAPs. Table 3 shows the five proteins with the highest increases and decreases in fold change (FC) in abundance, along with additional proteins that are relevant to the search for disease biomarkers. Notable DAPs included changes in the amount of a2 macroglobulin, hemopexin, albumin apoB100, complement C3 and ice‐structuring protein type 4 which were lower in CMS than PD, while nucleoside diphosphate kinase, b‐enolase, creatine kinase, and fructose biphosphate aldolase exhibited higher levels in PD compared to CMS.

**TABLE 3:**
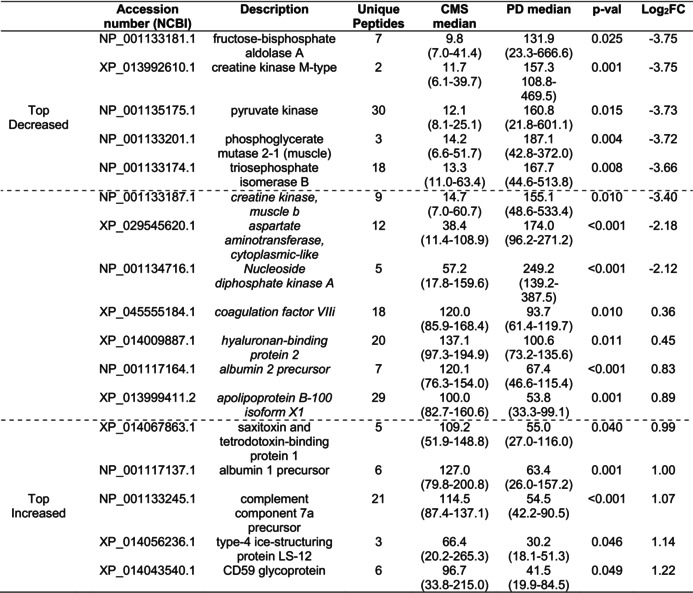
The five most decreased and five most increased differentially abundant proteins in comparison of samples from CMS to PD Atlantic salmon, including in italics and between the dotted lines further proteins of potential biomarker applications.

Finally, the comparison of Atlantic salmon serum proteome between samples from fish with PD and healthy samples is depicted in the volcano plot (Figure S3) and heatmap (Figure 3C). The five proteins with the greatest increasing FC and the greatest decreasing FC in abundance are given in Table 4, along with further proteins that had relevance to the search for biomarkers of disease. Several proteins that changed in abundance in CMS also changed in PD, but to a greater extent, with greater log_2_FC determined. Additional proteins were found to change specifically in PD, including increases in b‐enolase, fructose biphosphate aldolase, creatine kinase, pyruvate kinase, and a2 macroglobulin. On the other hand, there were reductions in PD for complement C4‐B, apo A‐II, apoB100, albumin and vitamin K‐dependent protein C.

**TABLE 4:**
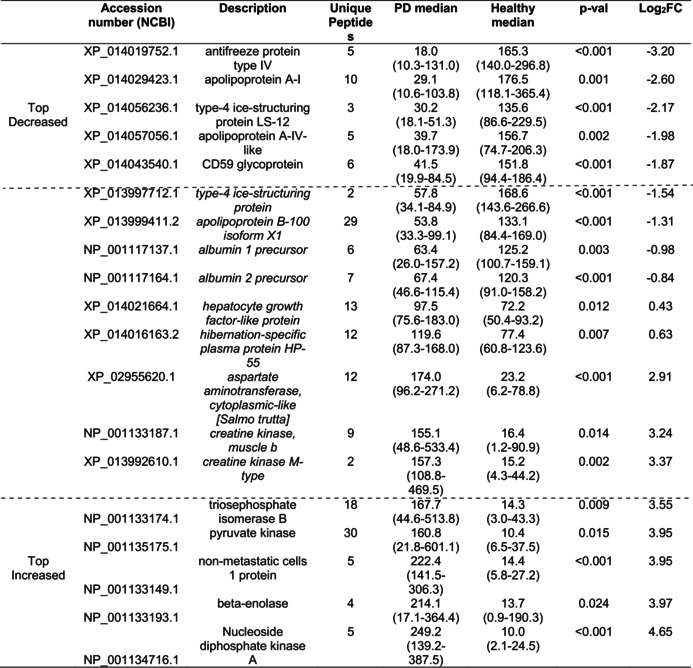
The five most decreased and five most increased differentially abundant proteins in comparison of samples from PD to healthy Atlantic salmon, including in italics and between the dotted lines further proteins of potential biomarker applications.

When all protein abundance values were analysed by PCA (Figure 5), PD samples formed a separate cluster that did not overlap with the healthy or CMS groups, whereas the CMS cluster showed considerable overlap with the healthy cluster. Assessment of individual points revealed four CMS samples that were outside the healthy cluster and separate from the PD cluster (CMS4, CMS6, CMS7 and CMS9).

Functional analysis of the DAPs found in comparing healthy to either CMS or PD indicates (Table 5) that most proteins (*n* = 15) are associated with innate immunity, particularly the acute phase response. This is followed by proteins with enzymatic function in energy metabolism (*n* = 8), which were particularly elevated in PD, and proteins related to coagulation functions (*n* = 7) and lipid metabolism/transport (*n* = 6). Additionally, some proteins could be related to heart function (*n* = 2) and temperature‐related functions (*n* = 3) while a group with miscellaneous functions was also identified (*n* = 22). Notably, some proteins are in multiple functional groups; for example, ApoAI and ApoAIV are included in both the innate immunity and lipid metabolism/transport categories.

**TABLE 5:** Classification of differentially abundant proteins by function assessed by the protein GenInfo Identifier (GI) accession numbers belonging to DAP from all three comparisons was inputted into The Database for Annotation, Visualisation and Integrated Discovery (DAVID) (Sherman et al. 2022). The direct Gene Ontology (GO) terms were assessed to understand gene enrichment with respect to biological process (BP).

Function	CMS vs. healthy	CMS vs. PD	Healthy vs. PD
Innate immunity including acute phase response	Ig kappa regions Plasma protease inhibitor C1 hemopexin Apoa1 Complement H Complement B Complement C1q Pentraxin	Hemopexin Albumin α2‐Macroglobulin complement C2 complement C3 Inter α‐trypsin inhibitor H	Albumin complement D α2‐Macroglobulin complement C4‐B Serotransferrin Inter α‐trypsin inhibitor H
Energy metabolism		Creatine kinase Triosephosphate isomerase Fructose biphosphate aldolase Pyruvate kinase Phosphoglycerate mutase aspartate transaminase β‐Enolase glycerol 3‐Phosphate dehydrogenase	Creatine kinase Triosephosphate isomerase Pyruvate kinase β‐Enolase
Coagulation	α‐Fibrinogen β‐Fibrinogen γ‐Fibrinogen Coagulation factor Viii Hyaluronan‐binding protein	Coagulation factor Viii Hyaluronan‐binding protein Plasminogen	Plasminogen
Lipid metabolism/transport	Apoaiv Apoa1 Apoc1 Apo AII	Apob100	Apoa1 Apoaiv Apob100 Apoeb
Cardiac function	Nucleoside diphosphate kinase Apo AIV	Nucleoside diphosphate kinase	Nucleoside diphosphate kinase
Temperature‐related	Ice structural protein Antifreeze protein Hibernation‐specific plasma protein HP‐55‐like	Ice structural protein	Antifreeze protein Ice structural protein Hibernation‐specific plasma protein HP‐55‐like

### 3.3 Validation of Proteomic Results

**FIGURE 4:**
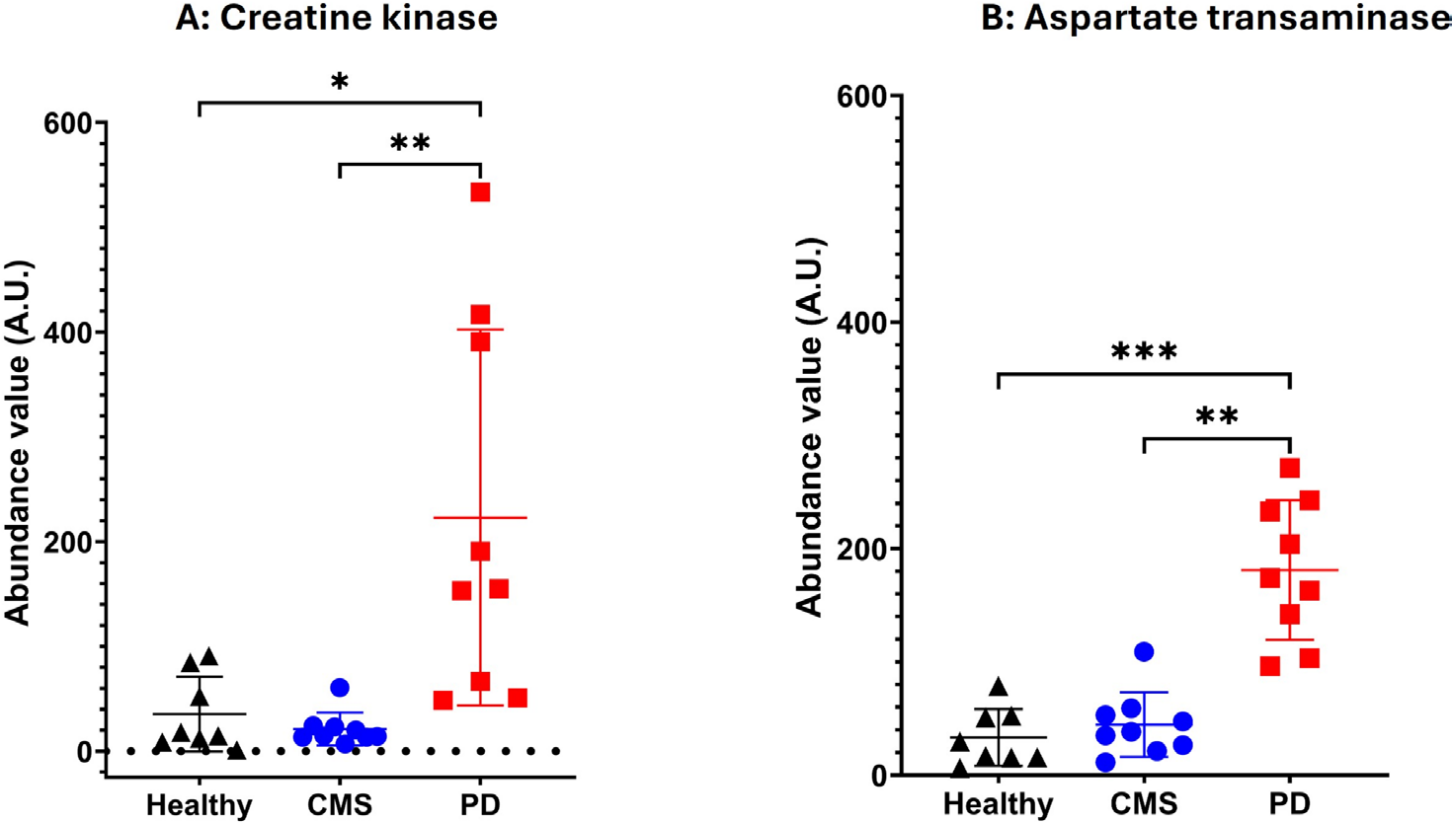
The abundance in TMT proteomics of CK (A) and (B) AST in serum from healthy Atlantic salmon (*n* = 8) and those with CMS (*n* = 9) or PD (*n* = 9) as determined by TMT‐proteomics assay. Significant differences between groups were determined by Kruskal–Wallis and Dunn's multiple comparison test. *p ≤ 0.05; **p ≤ 0.01; ***p ≤ 0.001.

Comparison of the TMT proteomic results to independent assay systems for specific proteins was used to validate the proteomic findings. CK assessed as a DAP by TMT proteomics and compared to the biochemical enzymic assay (Figure 4) of the Atlantic salmon serum samples demonstrated similar significant increases in PD compared to healthy samples. The proteomic results also showed that the PD values were significantly greater in PD serum samples than in CMS serum samples, although the enzyme assay did not yield a significant difference in this comparison.

**FIGURE 5:**
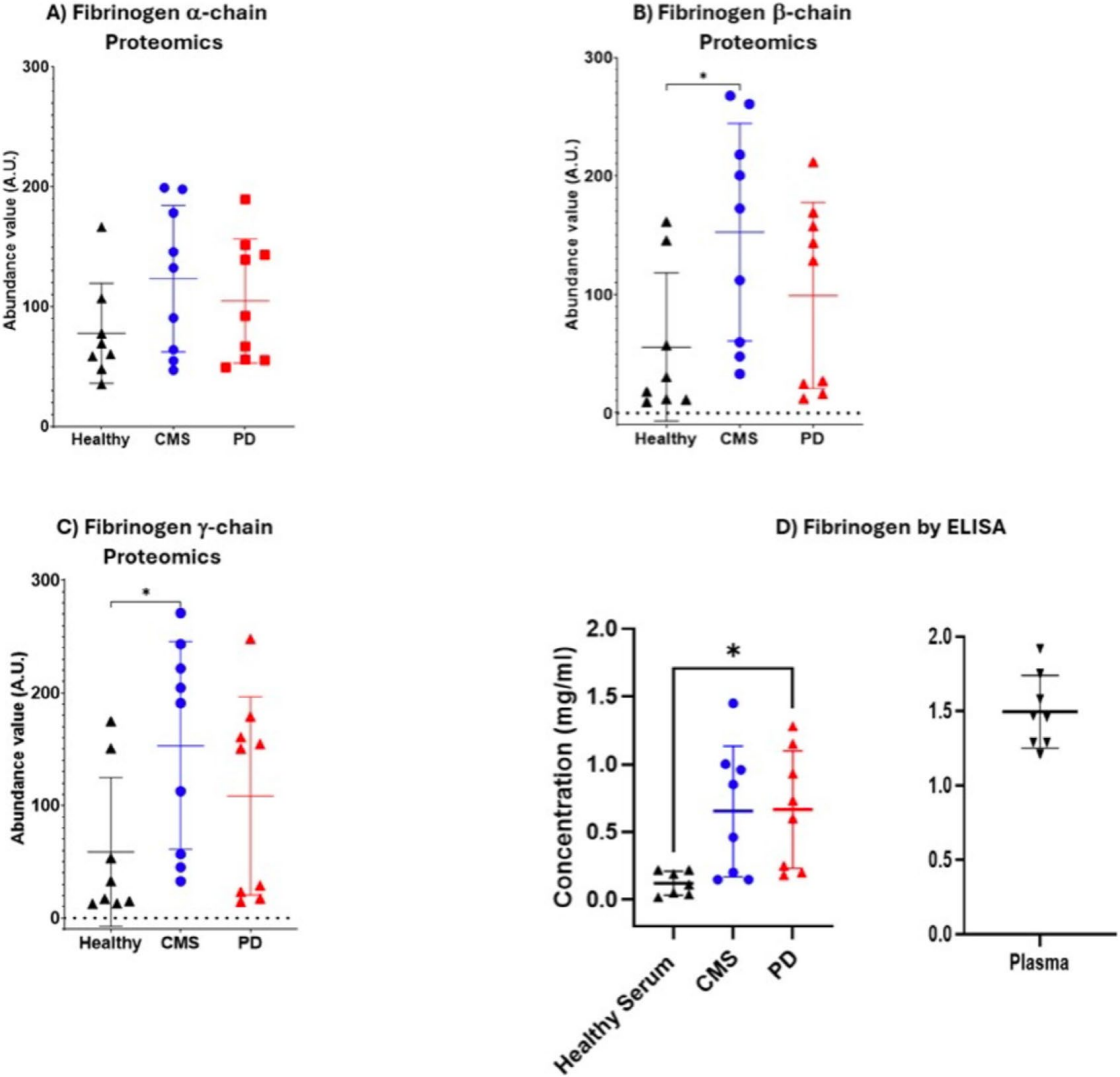
The abundance in TMT proteomics of (A) α‐fibrinogen, (B) β‐fibrinogen and (C) g‐fibrinogen from healthy Atlantic salmon (*n* = 8), and those with CMS (*n* = 9) or PD (*n* = 9) as determined by TMT‐proteomics assay. (D) The concentration of serum fibrinogen determined by ELISA with antiserum to trout fibrinogen in serum from healthy Atlantic salmon (*n* = 7) and those with CMS (*n* = 8) or PD (*n* = 8) in comparison to the concentration of fibrinogen in healthy plasma (*n* = 8). Significant differences between groups determined by Kruskal–Wallis and Dunn's multiple comparison test i.e., *p(0.05).

The assessment of AST as a DAP (Figure 5) revealed significantly higher levels in PD serum compared to CMS and healthy sera. However, while a similar trend was observed in AST enzyme activity, there were no significant differences between the groups.

Comparison of albumin 1 precursor and albumin 2 precursor as DAP by TMT proteomics revealed that their abundances in PD samples were significantly lower than in CMS or healthy samples for both isoforms, but there was no significant difference between groups in ALB concentration by biochemical analysis (Figure S4), as it does not differentiate between isoforms.

**FIGURE 6:**
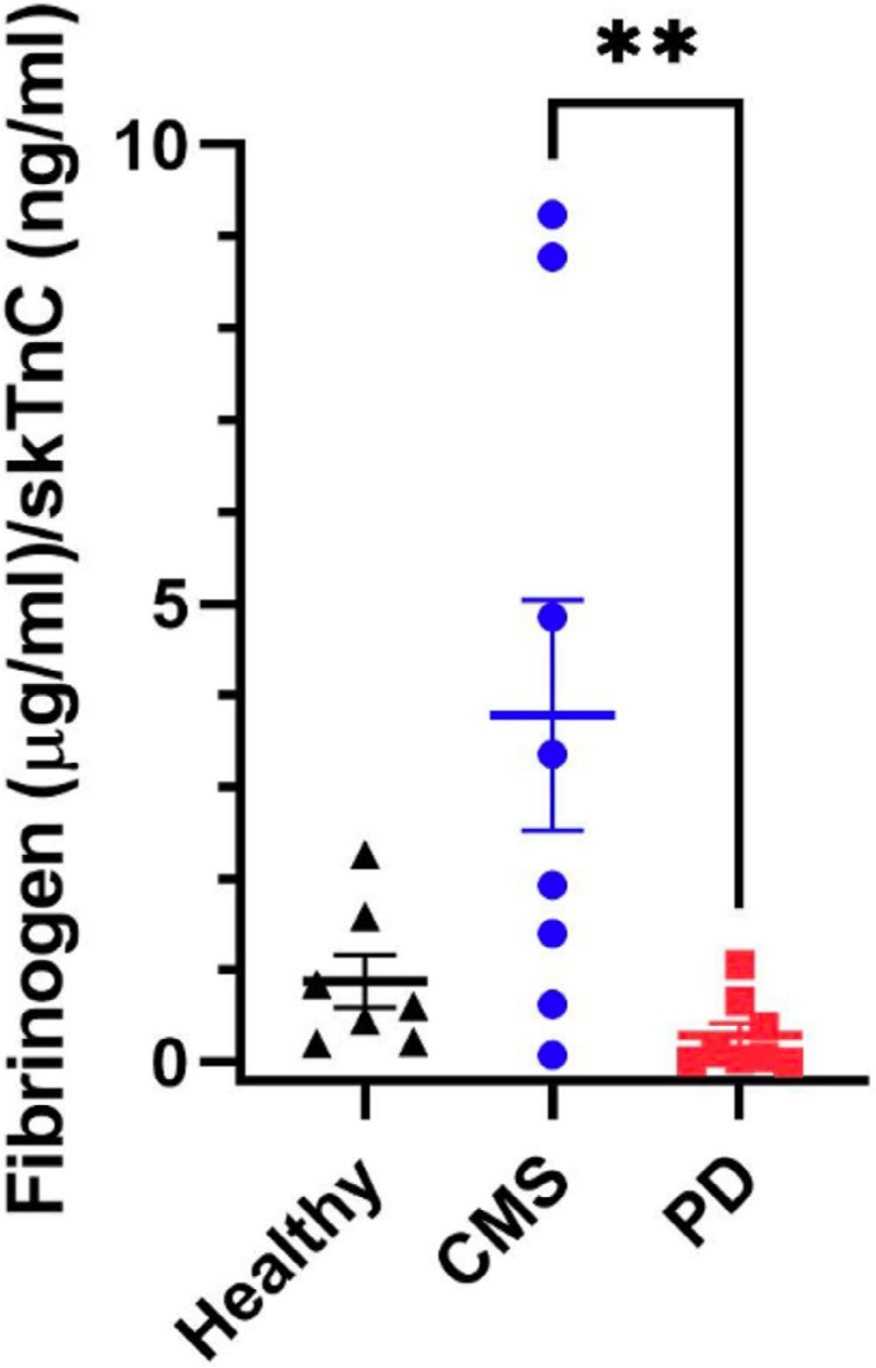
The ratio of fibrinogen concentration (mg/mL) to skeletal troponin concentration (ng/mL) in serum from healthy Atlantic salmon (*n* = 7) and those with CMS (*n* = 8) or PD (*n* = 8). Significant differences between groups determined by Kruskal–Wallis and Dunn's multiple comparison test i.e., *p(0.05).

**FIGURE 7:**
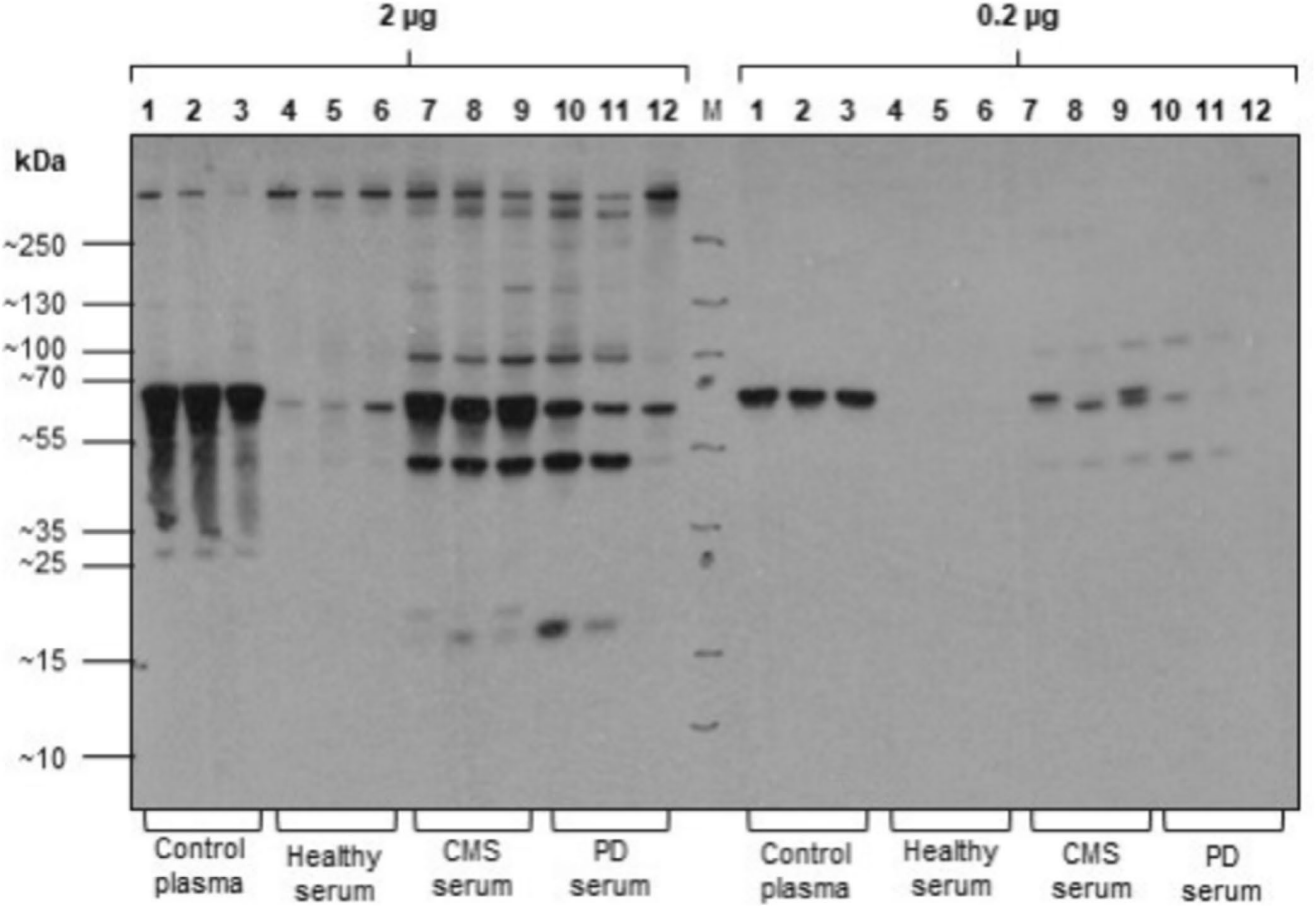
Western blot of Atlantic salmon serum from fish from healthy farms and those from farms with outbreaks of CMS or PD (*n* = 3 from each group), with total protein loading at 2.0 or 0.2 mg of protein. Control material of three Atlantic salmon plasma samples was included in lanes 1–3. The blot was developed with a rabbit antibody to trout fibrinogen (Life Diagnostics, West Chester, USA) as the first antibody at a dilution of 1:40,000 and goat anti‐rabbit IgG conjugated to HRP (AbCam #ab6721) at a dilution of 1:5000.

The assessment of fibrinogen for validation of the TMT proteomics entailed comparison of the three forms of fibrinogen identified in the proteomic analysis to the fibrinogen concentration determined by immunoassay using an antibody targeting total fibrinogen of trout. a‐Fibrinogen was identified in all groups of serum samples but showed no significant differences in abundance, while b and g‐fibrinogens showed significantly higher abundances in the CMS samples than in healthy samples, but no difference to the PD samples (Figure 6). Using the ELISA based on a specific antibody to trout fibrinogen, the concentration of Atlantic salmon fibrinogen gave, in comparison with healthy serum, a significant increase in samples from salmon with PD (*p* = 0.023) and a tendency towards significance in serum from fish with CMS (*p* = 0.053) was found (Table 6). One of the healthy serum samples was considered to be an outlier with a concentration of 1.12 mg/mL compared with mean and SD of 0.12 ± 0.09 (Table 6) for the remaining samples and was removed from analysis (Grubbs test *z* = 2.41, *p* < 0.01 Graphpad Prism). Calculation of the ratio between fibrinogen and skTnC (fibrinogen/skTnC) revealed (Figure 7) that the ratio was significantly greater (*p* < 0.01) in CMS (3.8 ± 3.6) than in PD (0.28 ± 0.38), although there was no significant difference between the ratio in CMS and healthy (0.88 + 0.77) samples. The western blot, using the antibody to trout fibrinogen, of serum from all groups compared to controls consisting of Atlantic salmon plasma samples containing normal levels of fibrinogen (Figure 7), revealed a main band at 70 kDa in the plasma sample, while healthy serum samples had minimal reaction at this or any molecular weight of protein especially at a loading of 0.2 mg of total protein, while in CMS, samples at 0.2 mg loading gave bands of a similar Mw. With the higher loading of 2 mg total protein, the plasma and CMS and PD serum samples exhibited more intense bands in response to the anti‐trout fibrinogen.

**TABLE 6:** Fibrinogen in serum and plasma from Atlantic salmon.

Group	Fibrinogen mg/mL (Mean ± SD)	Fibrinogen/skcTnC ratio**
Healthy serum (*n* = 7)*	0.12 ± 0.09^a^	0.88 ± 0.77^a,c^
CMS serum (*n* = 8)	0.65 ± 0.48^a,b^	3.78 ± 3.56^a,b^
PD serum (*n* = 8)	0.67 ± 0.43^b,c^	0.29 + 0.39^a,c^
Healthy plasma (*n* = 8)	1.49 ± 0.24^d^	N/A

*Note:* a,b,c No significant difference between groups.

* An outlier at 1.12 mg/mL was omitted from the healthy group.

** Fibrinogen as μg/mL compared to skTnC in ng/mL.

Apolipoproteins identified as DAP in comparisons between groups particularly for apolipoprotein A1, which was significantly lower in both CMS and PD samples compared to healthy samples (Figure S5A). ApoB‐100 was significantly lower in PD than in CMS and healthy groups (Figure S5B). Furthermore, samples from Atlantic salmon with PD exhibited significantly lower ApoA‐IV like abundance compared to healthy samples (Figure S5C). Assessing the lipoprotein concentration via their cholesterol content of HDL‐chol and LDL‐chol did not reveal any significant differences between groups in HDL‐chol but showed a significant decrease in LDL‐chol in the PD group. However, the total cholesterol had significant reductions in cholesterol in both the CMS and PD samples (Figure S5D).

Further examination of the proteomic data from the comparison of the CMS versus healthy samples identified proteins that may have potential as biomarkers for CMS in being able to differentiate between healthy and CMS groups while also being differentiated from PD infection. Notable among such proteins that differed in CMS compared to healthy included reductions in antifreeze protein type IV and type‐4 ice‐structuring protein and increases in coagulation factor VIIi, hibernation‐specific plasma protein HP‐55‐like, hyaluronan‐binding protein 2, and hepatocyte growth factor‐like protein (Figure S6). For those proteins that decrease in abundance in CMS, the reduction is greater in PD. Conversely, for the proteins that increase in abundance in CMS, coagulation factor VIIi, hibernation‐specific plasma protein HP‐55‐like, and hyaluronan‐binding protein 2 exhibited greater increases than in either PD or healthy samples, although not all changes observed were significant.

## 4 Discussion

This study applied proteomics and serum biomarkers for assessing Atlantic salmon health, in order to enhance diagnosis of CMS and PD and to differentiate between the virus infections. This approach aims to enhance diagnostic capabilities and improve health monitoring practices for Atlantic salmon in aquaculture by obviating the need to sacrifice salmon and send tissue samples to laboratories for histopathology. To fully utilise serum biomarkers in the future to enable rapid on‐farm diagnosis of cardiomyopathy inducing viral diseases of salmon, a challenge identified in this study will be in distinguishing CMS from PD, as both conditions induce similar changes in serum proteins. One fascinating finding of the study was that serum fibrinogen was identified as a potential biomarker for use in salmon health assessment. The conventional wisdom is that unlike plasma, serum should contain minimal concentrations of fibrinogen, which is expected to be converted to fibrin and then removed by centrifugation. Here, identification of significantly raised concentrations of fibrinogen in serum from salmon with both CMS and PD not only indicates their use in disease monitoring but understanding this phenomenon may provide insight into the pathophysiology of these viral cardiomyopathies.

In regard to the biomarker and proteomic results comparing PD and CMS, a more pronounced deviation from healthy serum profiles occurs in the former, whereas CMS presents only moderate alterations, as exemplified by the cTnC and skTnC concentrations in disease groups, complicating the differential diagnosis. The quantitative TMT‐proteomics of this investigation revealed several further proteins that demonstrated increases in PD resembling the observed changes in cTnC and skTnC, suggesting they could serve as alternative biomarkers for monitoring PD, but for many of these DAPs, the relative increase in CMS was lower than in PD, similar to the change in cTnC. While these proteins may be useful for the diagnosis of CMS, their potential inability to differentiate between CMS and PD, either in early or late stages where low levels of biomarkers are measured, may limit their diagnostic value. The ideal biomarker for CMS would be a serum protein responding to CMS exhibiting a greater response than that observed in PD infection. This study successfully identified a small number of proteins with such potential and therefore focused on those with distinctive responses. The data generated has also enabled additional insights into the pathophysiology of Atlantic salmon during outbreaks of PD and CMS.

A limitation of this study is that, due to practical constraints associated with sample collection under commercial aquaculture conditions, it was not possible to obtain tissue samples from the same fish used for serum analysis. Disease classification was instead based on population‐level clinical examination at the time of sampling, supported by qPCR results and histopathology from moribund fish within the same population. Fish included in the CMS experimental group were sampled from a population undergoing a clinical CMS outbreak, with clinical diagnosis supported by qPCR positivity. These fish also presented with moderately elevated serum cTnC (mean ± SD = 58 ± 28 ng/mL), which was 1–2 orders of magnitude above that of the healthy group. In contrast, the PD group was sampled from a population undergoing a clinical PD outbreak, defined by qPCR positivity for SAV in cardiac tissue from moribund fish. These fish exhibited markedly elevated serum cTnC (mean ± SD = 1937 ± 520 ng/mL), 3–4 orders of magnitude above the healthy group. Finally, the control group consisted of clinically healthy fish, with an average serum cTnC concentration of 0.42 ± 0.27 ng/mL. For the proteomics analysis, serum samples (*n* = 9 per group) were selected from CMS, PD outbreaks and healthy groups. While histological confirmation was not possible for individual fish, the clinical examination in combination with qPCR results and biochemical profiling provided sufficient evidence to consider these samples representative of the affected populations. This limitation highlights the challenges of field‐based sampling and underscores the need for accessible, serum‐based diagnostic tools for more accurate and timely disease identification in Atlantic salmon.

Following the TMT‐proteomic study of the serum samples, a total of 674 proteins were identified. After removing those with only 1 unique peptide and applying a 5% FDR, 57 proteins were classified as DAPs for comparisons between the experimental groups. The heat maps and volcano plots showed significant differences in DAPs between the groups. The PCA provided an overview of the results, indicating that while PD serum samples were distinctly separated from the other groups, there was an overlap between CMS and healthy. The functional analysis of the proteins that differ during infection (Table 6) identified the innate immune response, including the acute phase reaction and complement cascade, as the principal metabolic reaction to the viral infections, with 15 DAPs being related to these pathways. However, while the proteins identified as related to the acute phase response, such as serotransferrin, hemopexin, plasma protease inhibitor C1 and albumin and though their abundances differed in abundance between groups, they were not among the five most increased or decreased proteins in TMT‐proteomics. Other possible acute phase proteins of Atlantic salmon, such as serum amyloid A and haptoglobin, were not identified, which may be attributed to several factors. One possible explanation is that the concentrations of these proteins are below the sensitivity of the TMT‐based method, which may fail to detect low‐abundance proteins, especially in complex serum samples. Alternatively, their increase in concentration is transient, and at the time of sampling, these proteins could have already been cleared from circulation, making their detection more challenging (Bayne and Gerwick 2001). Therefore, a combination of these factors could explain the lack of detection of these APPs in our dataset. Eight DAPs associated with energy metabolism were identified, including CK, pyruvate kinase and AST. These proteins were notably elevated in the comparison between samples from fish with PD and those with control and CMS. This finding aligns with the muscle pathology observed as a result of cellular damage in PD‐infected fish, where cytoplasmic enzymes are released into the bloodstream (Braceland et al. 2013). Additionally, six DAPs identified were apolipoproteins derived from HDL, VLDL, LDL or chylomicrons, indicating their role in lipid transport. Disruption to the apolipoproteins is a known feature of infections in terrestrial animals and is particularly noted for their role in affecting HDL (Ansell et al. 2005), so this change in lipid transport proteins and a reduction in total serum cholesterol indicate that the viral infections have an impact on lipid transport and metabolism in Atlantic salmon. Other six DAPs were identified as coagulation‐related proteins, likely associated with fibrin clot formation, a recognised pathology of CMS, and also present in PD (Garseth et al. 2018). Two DAPs, nucleoside diphosphate kinase (Lutz et al. 2001) and apoA‐IV (Qu et al. 2019), have been linked to cardiac function in human clinical assessments. It is also noteworthy that apoA‐IV is also considered to be a major apolipoprotein in chylomicrons (Kohan et al. 2012), formed in the intestine to transport lipid to the liver and other tissues. A reduction in this apolipoprotein may indicate reduced intestine function during infections. Two further DAPs, ice‐structuring protein (Di Natale et al. 2019) and antifreeze protein, share structural similarities with ApoA‐I (Mao et al. 2018) and are involved in minimising the effects of a cold environment. It remains to be established whether the changes in their abundance were due to infection or sea temperature at the time of sample collection.

In validation of the results of the relative quantitative proteomics using TMT labelling, the validity of results was confirmed by proteomic results for CK and AST (Figure 4), comparing closely with their enzymic activity (Table 1) and the proteomic analysis was also able to show statistically different results between groups. High‐sensitivity techniques such as proteomics are known to detect such variations with greater precision (Aebersold and Mann 2016). In further validation of the proteomics, the difference between groups for serum fibrinogens was further explored. Examining changes in the fibrinogen abundance by quantitative proteomics showed that while a‐fibrinogen showed no differences between groups, both b and g fibrinogen showed significant increases in the CMS compared to the healthy group (Figure 6A–C). The use of the ELISA to determine the serum total fibrinogen concentration revealed significant and close to significant increases of PD and CMS samples respectively compared to healthy serum (Figure 6D). Although the fibrinogen concentration in the CMS serum was not significantly greater than the healthy serum, with *p* = 0.053, there is a clear tendency for significance and, with the supporting evidence from the Western blot, it can be concluded that serum fibrinogen is raised in CMS. For use in differential diagnosis of CMS from PD, the calculation of the ratio of fibrinogen to skTnC concentration revealed a significant increase in the ratio for CMS compared to skTnC. This results from the very large concentration of the latter in salmon with PD, and in this study, the calculation of this ratio is the most promising biomarker‐based analysis to differentiate between these diseases in field samples.

Furthermore, the Western blot of the samples from the serum groups, when compared to plasma, showed that when 0.2 mg of total protein was loaded, plasma revealed a single band of 70 kDa, which would correspond to the b‐fibrinogen subunit. This protein was absent from healthy serum, while it was clearly greater in CMS than PD samples. The observation of fibrinogen in serum was unexpected, as the coagulation of a blood sample in serum preparation would be expected to convert the fibrinogen into the fibrin clot (Pieters and Wolberg 2019) that is then separated by centrifugation. However, in the case of CMS, where internal fibrin formation is a known pathological effect of the disease, there could be endogenous fibrinolytic activity due to the enzymic action of plasmin on the degradation and removal of the fibrin clot (Pieters and Wolberg 2019). The fibrinogen chains detected by the proteomic investigation may be fibrin degradation products, including D‐dimer, which are established diagnostic biomarkers in human investigation (Luo and Roan 2017). With sample loading of 2 mg of total protein, additional bands, especially in the CMS and PD serum samples, were observed at 90 and 50 kDa. These bands may represent a and g fibrinogen, respectively, while a band of low intensity was seen at 130 kDa in some of these samples. When comparing the staining intensity of Atlantic salmon plasma samples on the Western blot to the CMS serum samples at the 0.2 mg of total protein loading, there was still a considerable amount of this protein present. This indicates its potential as a valuable biomarker for CMS, as the protein showed a more intense signal in the Western blot for CMS samples compared to both healthy and PD samples. It is possible that measuring serum fibrinogen in Atlantic salmon is the equivalent of the assay of fibrin degradation products, such as D‐dimer, which are established tests in detecting clot formation in human circulation. However, an attempt to detect Atlantic salmon D‐dimer using an antibody to human D‐dimer was not successful. It is possible that measurement of total fibrinogen would be a viable biomarker of CMS and be able to differentiate between healthy serum and that of Atlantic salmon affected by outbreaks of this disease. For differentiating CMS from PD, a combination of biomarker results could be used; an increase in fibrinogen accompanied by very high concentrations of cTnC and skTNC would be indicative of PD, while an increase in fibrinogen with only a slight increase in cTnC and no increase in skTnC may be indicative of CMS. Indeed, the significant difference found between CMS and PD groups in the fibrinogen/skTnC ratio (Figure 6) may provide the most differentiation between these cardiomyopathies. Further investigation is needed to determine relative clinical decision levels for the combination of fibrinogen and skTnC for such interpretation.

In comparison of the changes in apoA1 and apoB100 to changes in HDL‐cholesterol and LDL‐cholesterol, the results of the lipoprotein‐related cholesterol did not indicate a disease‐related alteration in their concentrations of cholesterol, and the tests for HDL‐cholesterol and LDL‐cholesterol would not be suitable for use as biomarkers in CMS or PD. To assess whether ApoA1 could be a negative biomarker for either PD or CMS, an immunoassay using an antibody against or that cross‐reacts with Atlantic salmon ApoA1 would be required.

Further examination of DAPs identified potential biomarkers for CMS, including antifreeze protein type IV and LS‐12 ice‐structuring protein, which exhibited negative responses in CMS samples but showed larger changes in PD samples. This suggests that while these proteins may be relevant for CMS, their greater alterations in PD limit their specificity for distinguishing between the two diseases. In contrast, coagulation‐related proteins, coagulation factor VIIi and hyaluronan‐binding protein 2, were significantly more abundant in CMS than in PD. The hibernation‐specific plasma protein HP‐55‐like also showed significantly higher levels in CMS compared to healthy samples and was higher, though not significantly, than in PD. Notably, hyaluronan‐binding protein 2 is involved in the coagulation activation network, and its increased abundance, along with factor VIIi, may reflect the pathophysiology of fibrin clot formation in CMS. Additionally, HP‐55‐like, associated with temperature changes, could be influenced by sea temperature rather than infection status.

The quantitative TMT proteomic investigation has revealed many changes in DAP during outbreaks of PD and CMS. However, differential diagnosis using serum from fish with these infections is challenging, as the extensive pathology and proteome changes of PD closely resemble those in CMS, though often more pronounced in PD. This may be critical to the development and use of biomarkers for these viral diseases, especially when a PD outbreak is followed in the same Atlantic salmon pen population by a CMS outbreak, with the possibility that complete recovery from the PD outbreak has not occurred. Nevertheless, there are indications that proteins identified by the TMT‐proteomics may be useful as biomarkers of CMS, possibly not as a single ‘silver bullet’ diagnostic test but as a part of a profile of tests including cTnC and skTnC that by combination in AI‐generated algorithms, could be highly indicative of a CMS outbreak. In these categories can be included coagulation factor VIIi and hyaluronan‐binding protein 2. Proteins elevated in both CMS and PD, such as nucleoside diphosphate kinase A may also be of value as a biomarker. Significant changes between groups in proteins that are potentially affected by temperature, such as antifreeze protein type IV, Type‐4 ice‐structuring protein LS‐12 and hibernation‐specific plasma protein HP‐55‐like could be usable as negative biomarkers of CMS and PD. However, this would require the production of specific antibodies for immunoassay development, and it would be interesting to determine if their abundance changes with alteration to sea temperature. Future research should also confirm by histopathology the health/infection status of Atlantic salmon being sampled and seek to extend the assessment of the use of assays for the potential CMS biomarkers with specific antibodies.

In summary, the relative quantitative proteomic investigation of Atlantic salmon serum from healthy fish and those in CMS and PD outbreaks was used to identify 57 DAPs in comparison between the groups. Of these DAPs, the majority showed the largest changes relative to healthy in the PD samples, with CMS exhibiting intermediate changes between PD and healthy samples. While these may be of use in establishing biomarkers for the diagnosis of CMS, a main finding of the study is that serum fibrinogen was at a low level in healthy samples but showed a higher abundance in CMS than PD samples and may, in combination with troponin analysis, suggest potential biomarker approaches for differentiation between CMS and PD. Therefore, further studies are warranted to evaluate their diagnostic value in assessing cardiomyopathies in Atlantic salmon and the pathophysiology of fibrin formation and degradation during the disease process.

## Author Contributions

The authors take full responsibility for this article.

## Conflicts of Interest

The authors declare no conflicts of interest.

## Supporting information


**Table S1:** Samples used for TMT proteomics with sea temperature and date of sample collection with serum biochemistry data: concentrations of cardiac Tropnin C, skeletal muscle Troponin C and activities of creatine kinase and aspartate transaminase.
**Figure S1:** Volcano plot of DAPs between CMS and healthy samples (*N* = 34). The *x*‐axis represents the log2 fold change (Log_2_FC), indicating the magnitude of protein expression differences between the two groups, while the *y*‐axis displays the ‐log_10_ *p*‐value, reflecting the statistical significance of these differences. Proteins with significantly higher abundance in the CMS group are shown in red (Log_2_FC > 0.2, *p* < 0.05), and those with significantly lower abundance in the CMS group are indicated in blue (Log_2_FC < −0.2, *p* < 0.05). Proteins with no significant change are represented in grey (*p* ≥ 0.05 or Log_2_FC between −0.2 and 0.2). The horizontal dashed line denotes the threshold for statistical significance (*p* = 0.05), while the vertical dashed lines indicate the log2 fold‐change thresholds (±0.2).
**Figure S2:** Volcano plot of DAPs between CMS serum and PD serum samples (*N* = 66). The *x*‐axis displays the log2 fold change (Log_2_FC), indicating the level of protein expression differences between the groups, and the *y*‐axis represents the ‐log_10_ *p*‐value, showing the significance of these differences. Proteins with greater abundance in CMS serum are shown in red (Log_2_FC > 0.2, *p* < 0.05), while those with reduced abundance in CMS serum are marked in blue (Log_2_FC < −0.2, *p* < 0.05). Non‐significant proteins are indicated in grey (*p* ≥ 0.05 or Log_2_FC between −0.2 and 0.2). The dashed horizontal line marks the *p*‐value significance threshold (*p* = 0.05), and the vertical dashed lines mark the Log_2_FC cut‐offs (±0.2).
**Figure S3:** Volcano plot of DAPs between PD serum and healthy serum samples (*N* = 81). The *x*‐axis represents the log_2_ fold change (Log_2_FC), showing the extent of protein expression differences between the two groups, while the *y*‐axis indicates the ‐log_10_ *p*‐value, reflecting the statistical significance of these differences. Proteins with significantly higher abundance in PD serum are highlighted in red (Log_2_FC > 0.2, *p* < 0.05), and those with reduced abundance are highlighted in blue (Log_2_FC < −0.2, *p* < 0.05). Proteins without significant changes are shown in grey (*p* ≥ 0.05 or Log_2_FC between −0.2 and 0.2). The dashed horizontal line marks the *p*‐value significance cut‐off (*p* = 0.05), and the vertical dashed lines indicate the log_2_ fold‐change boundaries (±0.2).
**Figure S4:** (A) The abundance of Albumin 1 precursor in serum from healthy Atlantic salmon (*n* = 8) and those with CMS (*n* = 9) or PD (*n* = 9) as determined by TMT‐proteomics assay. (B) The abundance of Albumin 2 precursor in serum from healthy Atlantic salmon (*n* = 8) and those with CMS (*n* = 9) or PD (*n* = 9). (C) The concentration of serum albumin from healthy Atlantic salmon (*n* = 8) and those with CMS (*n* = 9) or PD (*n* = 9).
**Figure S5:** (A) The abundance of Apoprotein A‐1 in serum from healthy Atlantic salmon (*n* = 8) and those with CMS (*n* = 9) or PD (*n* = 9); (B) The abundance of Apoprotein B‐100 in serum from healthy Atlantic salmon (*n* = 8) and those with CMS (*n* = 9) or PD (*n* = 9); (C) The abundance of Apoprotein A‐IV in serum from healthy Atlantic salmon (*n* = 8) and those with CMS (*n* = 9) or PD (*n* = 9) as determined by TMT‐proteomics assay. (D) The concentrations of total cholesterol, triglyceride, HDL‐cholesterol and LDL‐cholesterol in serum from healthy Atlantic salmon (*n* = 8) and those with CMS (*n* = 9) or PD (*n* = 9) as determined biochemical analysis.
**Figure S6:** Differentially abundant proteins of relevance to biomarkers of CMS and PD, (A) Nucleoside diphosphate kinase A; (B) Antifreeze protein type IV (C) type 4 ice‐structuring protein LS‐12 (D) Coagulation factor Viii (E) Hibernation‐specific plasma protein HP‐55‐like (F) Hyaluronan‐binding protein as determined by TMT‐proteomics assay.

**Table S2:** Proteomic Discoverer results from TMT analysis of serum from healthy (*n* = 8), CMS (*n* = 9) and PD (*n* = 9) Atlantic salmon (Excel file).

## Data Availability

The data that support the findings of this study are openly available in Proteomexchange at https://www.proteomexchange.org/, reference number 1‐20241030‐131842‐123970160.
