# Supplementary material for: Serum Biomarkers in Atlantic Salmon for Differential Diagnosis of Cardiomyopathy Syndrome and Pancreas Disease: Proteomic Identification of Serum Fibrinogen to Enhance Troponin Immunoassay as Optimal Diagnostic Approach
Source: J Fish Dis. 2025 May 29;48(11):e14151. doi: 10.1111/jfd.14151 (PMC12489428; doi:10.1111/jfd.14151)

**Supplementary information**

**Table S1** Samples used for TMT proteomics with sea temperature and date of serum collection with

serum biochemistry data: concentrations of cardiac Troponin C, skeletal muscle Troponin C, and

activities of Creatine Kinase and Aspartate Transaminase.

| **Sample Group/**  **Number** | **Date**  **collection**  **Month/year** | **Sea Temperature**  **(Month average)**  **^o^C** | **cTnC**  **ng/ml** | **skTnC**  **ng/ml** | **CK**  **IU/L** | **AST**  **IU/L** |
| --- | --- | --- | --- | --- | --- | --- |
| CMS1 | 03/22 | 7.8 | 16 | 115 | 272 | 796 |
| CMS2 | 03/22 | 7.8 | 46 | 261 | 88 | 244 |
| CMS3 | 02/22 | 7.5 | 71 | 398 | 48 | 476 |
| CMS4 | 02/22 | 7.5 | 91 | 2871 | 128 | 644 |
| CMS5 | 02/22 | 7.5 | 43 | 344 | 308 | 212 |
| CMS6 | 02/22 | 7.5 | 64 | 81 | 20 | 344 |
| CMS7 | 02/22 | 7.5 | 63 | 103 | 40 | 424 |
| CMS8 | 03/22 | 7.8 | 26 | 465 | 96 | 204 |
| CMS9 | 03/22 | 7.8 | 102 | 215 |  |  |
| PD1 | 07/21 | 11.5 | 1375 | 19799 | 672 | 1784 |
| PD2 | 07/21 | 11.5 | 2718 | 2499 | 172 | 1868 |
| PD3 | 07/21 | 11.5 | 1139 | 1908 | x | x |
| PD4 | 07/21 | 11.5 | 1917 | 2457 | 24 | 3604 |
| PD5 | 11/21 | 10.4 | 1851 | 29191 | 2528 | 13624 |
| PD6 | 11/21 | 10.4 | 2230 | 9110 | 3564 | 1568 |
| PD7 | 11/21 | 10.4 | 2187 | 31580 | 3400 | 4640 |
| PD8 | 11/21 | 10.4 | 2473 | 53183 | 52136 | 5644 |
| PD9 | 11/21 | 10.4 | 1541 | 6926 |  |  |
| Healthy1 | 02/22 | 7.5 | 0.28 | 211 | 0 | 100 |
| Healthy2 | 03/22 | 7.8 | 0.92 | 425 | 24 | 300 |
| Healthy3 | 07/21 | 11.5 | 0.42 | 118 | 0 | 336 |
| Healthy4 | 07/21 | 11.5 | 0.69 | 31 | 0 | 148 |
| Healthy5 | 03/22 | 7.8 | 0.24 | 539 | 556 | 1552 |
| Healthy6 | 03/22 | 7.8 | -0.09 | 1004 | 1044 | 416 |
| Healthy7 | 03/22 | 7.8 | 0.29 | 214 | 64 | 616 |
| Healthy8 | 03/22 | 7.8 | 0.53 | 1499 |  |  |
| *Healthy9 | 03/22 | 7.8 | 0.42 | 378 | 64 | 384 |

*Sample omitted from proteomic analysis, TMT protein labelling failed, pre-mass spectrometry

**Table S2**: Excel file of proteomic results

**Figure S1** Volcano plot CMS v Healthy


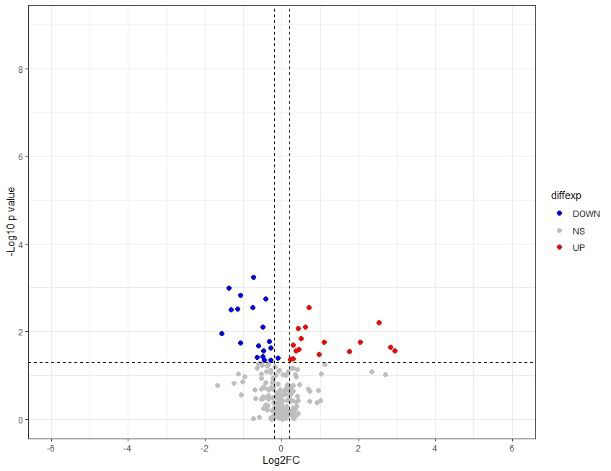


**Figure S2** Volcano plot CMS v PD


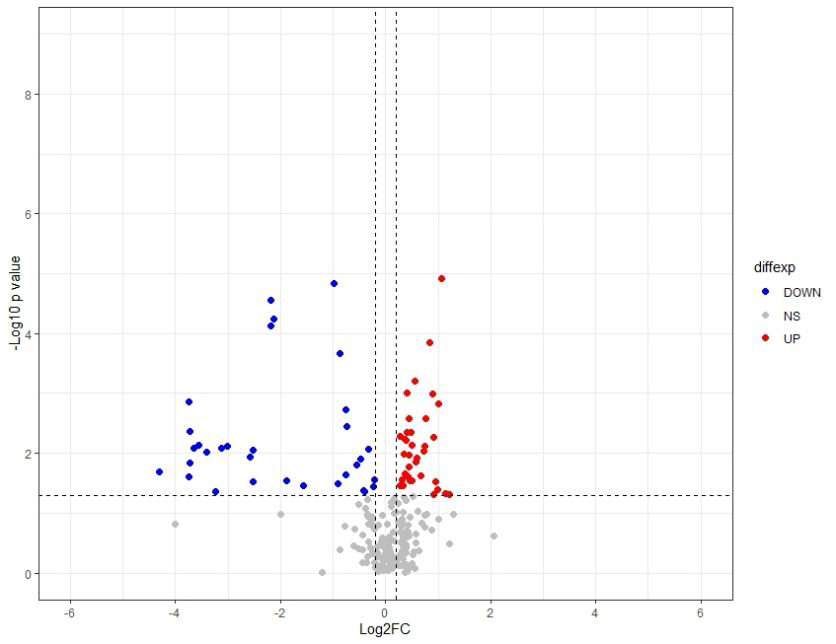


**Figure S3** Volcano plot PD v Healthy


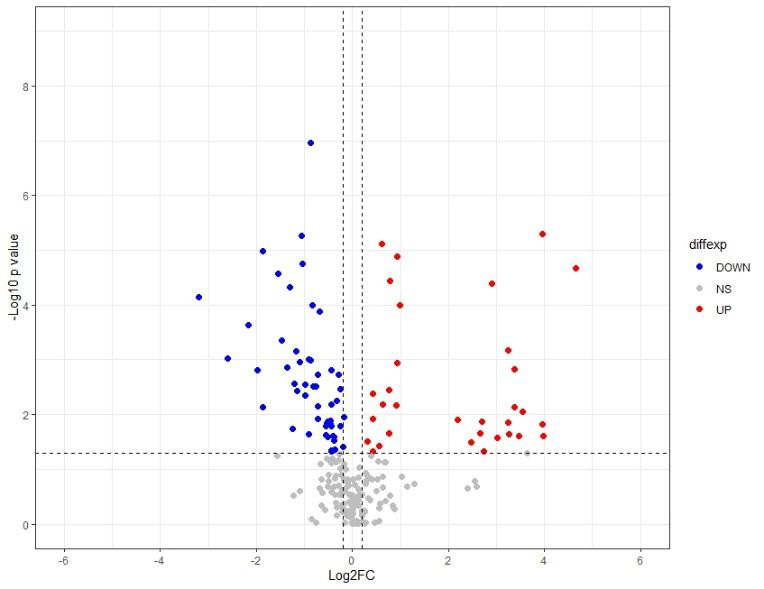


**Figure S4** Albumin


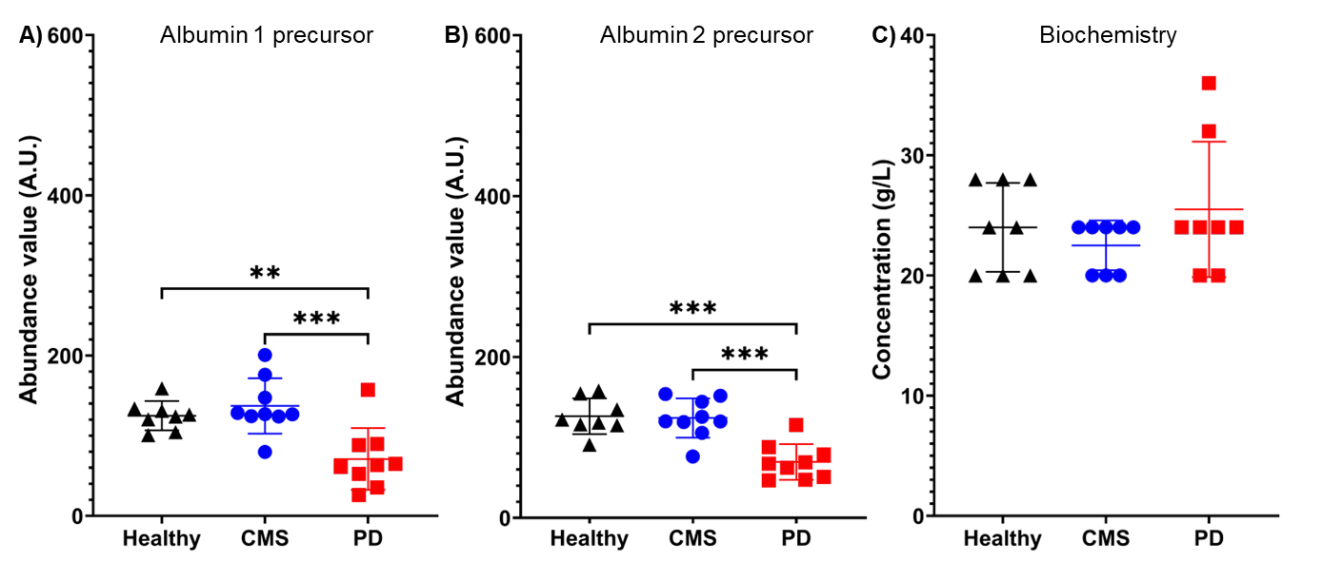


**Figure S5** Apolipoprotein and lipid measures


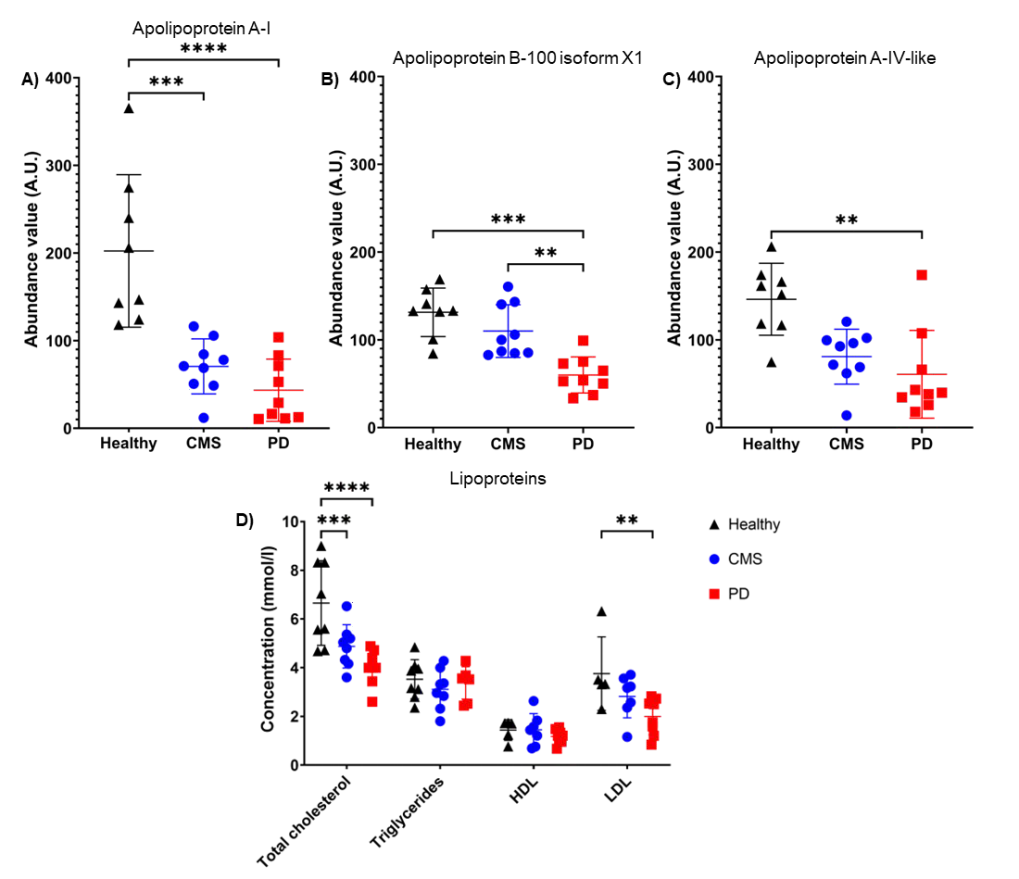


**Figure S6** Further differentially abundant proteins of relevance to biomarkers of CMS and PD


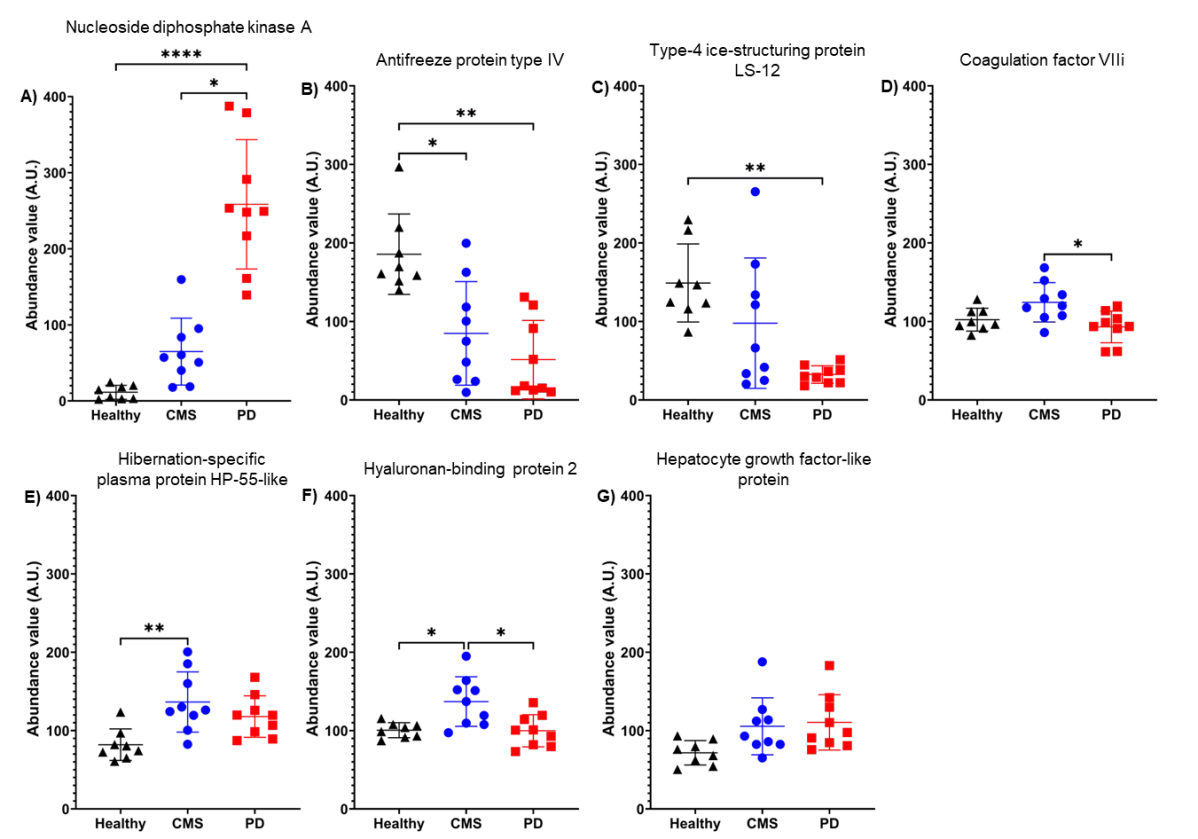

Supplement: Supplementary file 1 — Table S1: Samples used for TMT proteomics with sea temperature and date of sample collection with serum biochemistry data: concentrations of cardiac Tropnin C, skeletal muscle Troponin C and activities of creatine kinase and aspartate transaminase. Figure S1: Volcano plot of DAPs between CMS and healthy samples (N = 34). The x‐axis represents the log2 fold change (Log2FC), indicating the magnitude of protein expression differences between the two groups, while the y‐axis displays the ‐log10 p‐value, reflecting the statistical significance of these differences. Proteins with significantly higher abundance in the CMS group are shown in red (Log2FC > 0.2, p < 0.05), and those with significantly lower abundance in the CMS group are indicated in blue (Log2FC < −0.2, p < 0.05). Proteins with no significant change are represented in grey (p ≥ 0.05 or Log2FC between −0.2 and 0.2). The horizontal dashed line denotes the threshold for statistical significance (p = 0.05), while the vertical dashed lines indicate the log2 fold‐change thresholds (±0.2). Figure S2: Volcano plot of DAPs between CMS serum and PD serum samples (N = 66). The x‐axis displays the log2 fold change (Log2FC), indicating the level of protein expression differences between the groups, and the y‐axis represents the ‐log10 p‐value, showing the significance of these differences. Proteins with greater abundance in CMS serum are shown in red (Log2FC > 0.2, p < 0.05), while those with reduced abundance in CMS serum are marked in blue (Log2FC < −0.2, p < 0.05). Non‐significant proteins are indicated in grey (p ≥ 0.05 or Log2FC between −0.2 and 0.2). The dashed horizontal line marks the p‐value significance threshold (p = 0.05), and the vertical dashed lines mark the Log2FC cut‐offs (±0.2). Figure S3: Volcano plot of DAPs between PD serum and healthy serum samples (N = 81). The x‐axis represents the log2 fold change (Log2FC), showing the extent of protein expression differences between the two groups, while the y‐a [file JFD-48-e14151-s001.docx]
